# Lectin Pathway of Complement Activation Is Associated with Vulnerability of Atherosclerotic Plaques

**DOI:** 10.3389/fimmu.2017.00288

**Published:** 2017-03-16

**Authors:** Stefano Fumagalli, Carlo Perego, Rosalia Zangari, Daiana De Blasio, Marco Oggioni, Francesca De Nigris, Francesco Snider, Peter Garred, Angela M. R. Ferrante, Maria-Grazia De Simoni

**Affiliations:** ^1^Department of Neuroscience, IRCCS – Istituto di Ricerche Farmacologiche Mario Negri, Milan, Italy; ^2^Vascular Surgery Unit, Catholic University of Sacred Heart Medical School “A. Gemelli University Hospital” Foundation, Rome, Italy; ^3^Laboratory of Molecular Medicine, Department of Clinical Immunology, Section 7631, Rigshospitalet, Faculty of Medical and Health Sciences, University of Copenhagen, Copenhagen, Denmark

**Keywords:** cardiovascular diseases, atherosclerosis, complement system proteins, ficolin-2, vulnerable plaques

## Abstract

Inflammatory mechanisms may be involved in atherosclerotic plaque rupture. By using a novel histology-based method to quantify plaque instability here, we assess whether lectin pathway (LP) of complement activation, a major inflammation arm, could represent an index of plaque instability. Plaques from 42 consecutive patients undergoing carotid endarterectomy were stained with hematoxylin-eosin and the lipid core, cholesterol clefts, hemorrhagic content, thickness of tunica media, and intima, including or not infiltration of cellular debris and cholesterol, were determined. The presence of ficolin-1, -2, and -3 and mannose-binding lectin (MBL), LP initiators, was assessed in the plaques by immunofluorescence and in plasma by ELISA. LP activation was assessed in plasma by functional *in vitro* assays. Patients presenting low stenosis (≤75%) had higher hemorrhagic content than those with high stenosis (>75%), indicating increased erosion. Increased hemorrhagic content and tunica media thickness, as well as decreased lipid core and infiltrated content were associated with vulnerable plaques and therefore used to establish a plaque vulnerability score that allowed to classify patients according to plaque vulnerability. Ficolins and MBL were found both in plaques’ necrotic core and tunica media. Patients with vulnerable plaques showed decreased plasma levels and intraplaque deposition of ficolin-2. Symptomatic patients experiencing a transient ischemic attack had lower plasma levels of ficolin-1. We show that the LP initiators are present within the plaques and their circulating levels change in atherosclerotic patients. In particular, we show that decreased ficolin-2 levels are associated with rupture-prone vulnerable plaques, indicating its potential use as marker for cardiovascular risk assessment in atherosclerotic patients.

## Highlights

– Initiators of the lectin pathway of complement activation are expressed in atherosclerotic plaques.
– The activation of the lectin pathway is increased in vulnerable atherosclerotic plaques.
– Low Ficolin-2 plasma levels are associated with vulnerable atherosclerotic plaques.
– Ficolin-1 plasma levels are lower in symptomatic (vs. non-symptomatic) patients experiencing a transient ischemic attack.

## Introduction

Acute cardiovascular events, such as myocardial infarction and ischemic stroke, are associated with progression and rupture of unstable atherosclerotic plaques. Key to advance prevention of neurologic complications and to improve therapy is the early detection of rupture-prone atherosclerotic carotid plaques. Patients with stenosis above 70% may be eligible for surgical intervention, e.g., endoarterectomy (1). The degree of carotid stenosis is the only valid criterion currently used in clinical decision-making to assess the severity of atherosclerotic disease (2). However, stenosis alone is insufficient to reliably predict plaque instability. Unstable, or vulnerable, plaques may erode causing thromboembolic complications (3) and increased risk of transient ischemic attack and stroke recurrence (4, 5). Several morphologic studies using non-invasive imaging techniques have been published (6, 7); however, a clear-cut clinical definition of vulnerable plaques is not available; therefore, a meaningful surrogate of lesion instability detecting vulnerable plaques before symptomatology is needed.

Atherosclerosis depends on processes such as the oxidative modification of lipoproteins in the arterial walls, representing danger stressors that activate several components of the inflammatory response (8–10), including the complement system (11). The lectin pathway (LP) is a complement activation pathway showing a critical role in thrombosis (12). Moreover, the LP has pathogenetic functions in acute brain injuries such as ischemia or traumatic injury in experimental models (13–16) and in humans (13, 16–20), pathological conditions showing vascular impairment. The LP is activated by the initiator molecules mannose-binding lectin (MBL), ficolins (ficolin-1 or M-ficolin, ficolin-2 or L-ficolin, and ficolin-3 or H-ficolin), and collectin-11 (21–23). A few studies reported MBL involvement in atherosclerosis, though results are contrasting, supporting either an anti-atherogenic (24–28) or a pro-atherogenic (29–31) role. A few data are available on the LP involvement in plaque vulnerability (32), with still open questions.

In this study, we propose a histology-based approach that provides a quantitative assessment of plaque vulnerability. We selected four morphological parameters associated with plaque instability to set a vulnerability score. Finally, we investigated the association of LP initiators and activation with plaque vulnerability as indicated by the vulnerability score.

## Materials and Methods

### Patients

Plaques were obtained from 42 consecutive patients undergoing carotid endarterectomy. Patients were symptomatic (experiencing a transient ischemic attack (TIA)) and asymptomatic referring to department of Vascular Surgery at A. Gemelli Hospital during 2013–2015. Clinical data, obtained as part of standard patient care, and samples were retrospectively examined with the approval of the local ethical committee Board (Comitato Etico della Fondazione Policlinico Universitario A. Gemelli, reference number: 26089/16). Degree (%) of stenosis was assessed by echocolordoppler. Patients were stratified by 70, 75, 80, 85, and 90% stenosis, according to the ECST criteria (33) and guidelines of Italian Society of Vascular and Endovascular Surgery (34). Only patients with stenosis ≥70% were subjected to surgery and therefore included in this study (34). Patients were excluded from surgery and therefore from this study when the operation risks were considered too high.

Patients’ details, including therapies and comorbidities, are reported in Table 1. None of the patients had any autoimmune disease.

**Table 1:** **Patients’ details**.

Total patients = 37			%
Age mean ± SD		73.03 ± 7.42	
Gender (M/F)		31/6	84/16
Symptomatic (Y/N)		11/26	30/70
Stenosis (symptomatic)	70%	3 (2)	8 (67)
	75%	12 (2)	32 (17)
	80%	11 (3)	30 (27)
	85%	4 (1)	11(25)
	90%	7 (3)	19 (43)
Therapy with statins		33	89
Therapy with antiaggregants		35	95
No therapy		1	0.4
Smoking			
Current		6	16
Former		21	57
Never		10	27
Diabetes mellitus		16	43
Obesity		9	24
Hypertension		32	86
Dyslipidemia		34	92

### Sample Processing

Immediately after surgery specimens were snap-frozen. The plaques were examined macroscopically to identify the site of the maximal plaque thickening and then transversely dissected into two segments along the longitudinal axis. The plaque segment showing the largest plaque burden as determined by visual assessment was defined as the zero (0) segment (35). The rationale is that the segment of a plaque with the largest plaque burden is generally, where the largest lipid core and the more extensive inflammation are present. The 0 segment was the reference segment and the adjacent 1-mm-thick segments at both sides were called ±1, ±2, … The plaque region cut and used for histological analysis ranged from segments −1 to +1, being defined as the segments with the greatest plaque burden. Plaque segment cutting was performed on dry ice. Segments embedded in optimal cutting temperature medium were then transferred to a cryostate and cut in 20 μm-thick slices. Slices were laid on a gelatinized glass for subsequent histological stainings. Before stainings, sections were thawed by 5 min washing with 0.05M triphosphate buffer saline (TBS) at room temperature (RT) and then post-fixated by 15 min incubation with 4% paraformaldheyde.

Blood samples were obtained one day after surgery. Clotting and complement activation were prevented by collecting samples in 10 mM of ethylenediaminetetraacetic acid (EDTA). Plasma was processed at 2,000 g for 15 min at 4°C and stored −80°C before analysis.

### Histological Analysis

**Figure 1:**
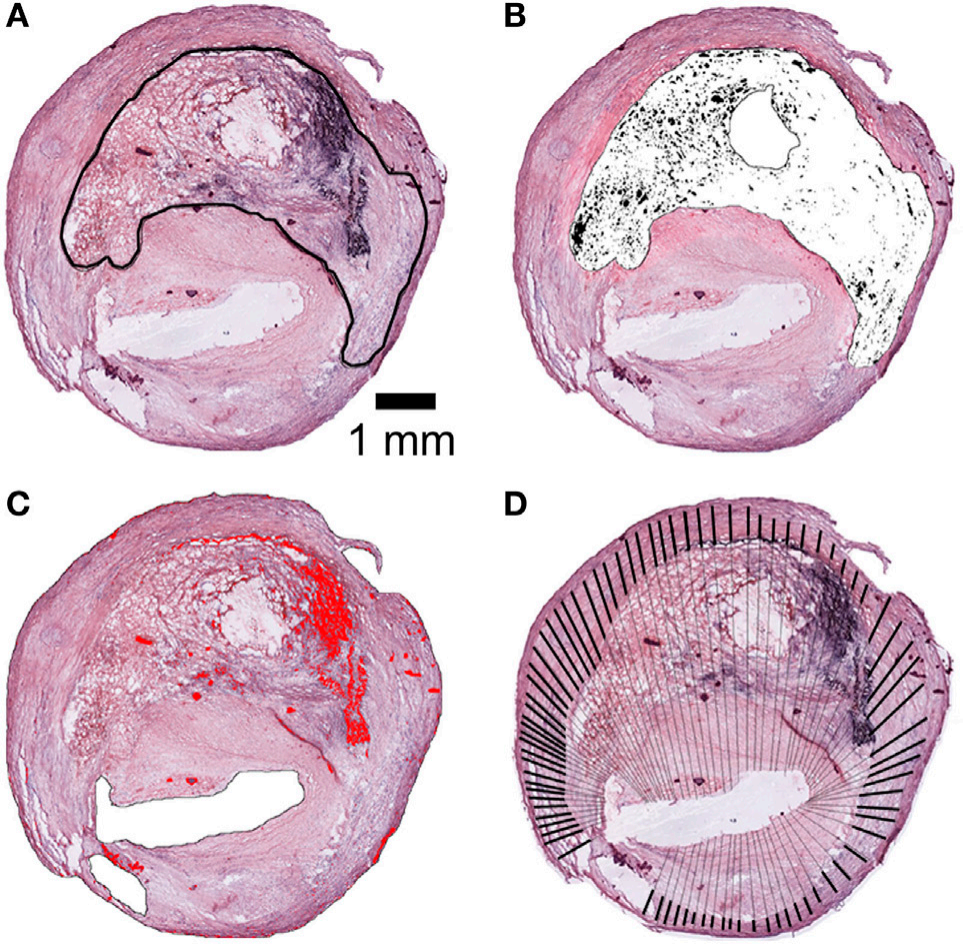
**Histological analysis of plaques**. **(A)** Lipid core area by manual outline tracing. **(B)** Cholesterol clefts area by software-based segmentation (black selection). **(C)** Hemorrhagic content by software-based segmentation (red selection). **(D)** Thickness of tunica media (heavy lines) and total thickness of tunica including infiltration of cellular debris and cholesterol (heavy and thin lines). The ratio between total and tunica media lengths corresponds to infiltrated content. Areas expressed in μm^2^ and normalized for total plaque area (μm^2^). Scale bar = 1 mm.

Hematoxyline and eosin (H&E) staining was performed according to standard protocols. H&E stained slices were acquired at 20× using a Nikon Eclipse Ni-E virtual stage microscope so to have complete stitching of the whole plaques at high resolution (1 pixel = 0.345 μm). A digital repository of stained plaques was created and used for subsequent quantifications. As a first evaluation, an investigator blinded to patients’ clinical data identified plaques with signs of rupture according to the classification proposed by Virmani et al. (36). For quantifications, images were scaled to microns. The area of intra-intimal lipid-rich core with hemorrhage and cholesterol clefts was calculated. Lipid core area was outlined on the plaque by manual operator selection (Figure 1A). In the lipid core area, cholesterol clefts were identified by gray scale thresholding and selected (Figure 1B). Hemorrhagic area was identified by digital color thresholding based on RGB pixel values (Figure 1C). Hemorrhagic content, lipid core, and cholesterol cleft areas were calculated as μm^2^ and expressed as percentage of total plaque area. Plaque thickness was measured manually along the traces outlined in Figure 1D. Total tunica thickness was measured at the thickest point at the border of the vascular lumen and the media layer, excluding adventitial cutting artifacts from analysis. The media thickness was measured from the intimo-medial interface to the external border. The intra-intima infiltrated content of cellular debris and cholesterol (from here on referred to as “infiltrated content”) was defined as the ratio between total tunica thickness and media thickness.

### Immunofluorescence and Confocal Analysis

After thorough washings with PBS 0.01M, 20-μm coronal sections were incubated with blocking solution (10% normal goat serum, 0.3% Triton X-100) for 1 h at RT and then with primary antibodies in the same solution overnight at 4°C. Primary monoclonal antibodies used were anti-human MBL (1:50, Abcam, USA), anti-human ficolin-3 (1:50, Hycult Biotechnologies, NL), and anti-human ficolin-2 and ficolin-1 (both 1:50) (37).

Sections were then incubated with biotinylated secondary antibody (1:200, Vector Laboratories) for 1 h at RT and followed by fluorescent signal coupling with streptavidine TSA amplification kit (fluorescein, Perkin Elmer, USA). Sections were then incubated with True-Black quencher (1:20 in 70% Ethanol, Biotium, USA) to quench non-specific fluorescent signal. Appropriate negative controls without the primary antibodies were performed. None of the immunofluorescence reactions revealed unspecific fluorescent signal in the negative controls (Figure S1 in Supplementary Material). Immunofluorescence was acquired using a scanning sequential mode to avoid bleed-through effects by an IX81 microscope equipped with a confocal scan unit FV500 with three laser lines: Ar–Kr (488 nm), He–Ne red (646 nm), and He–Ne green (532 nm, Olympus, JPN), and a UV diode. Three-dimensional images were acquired over a 10 μm *z*-axis with a 0.23 μm step size and processed using Imaris software (Bitplane, CH) and Photoshop CS2 (Adobe Systems Europe Ltd.). Exclusion images on single focal planes were obtained by Fiji software image calculator protocol (“subtract” function).

### Functional LP-Specific C3 Deposition Assay

A LP specific C3 deposition ELISA was performed to measure residual LP functional activity in patient plasma (38). A Maxisorp ELISA plate (NUNC™) was coated with 10 μg/mL mannan to test LP activation by MBL (38, 39), or 25 μg/mL acetylated bovine serum albumin (acBSA) to test LP activation by ficolins (21, 40) diluted in coating buffer (15 mM Na_2_CO_3_, 35 mM NaHCO_3_, pH 9.6) and incubated overnight at 4°C. Residual protein binding sites were saturated by incubating the plate with 1% BSA-TBS blocking buffer (0.1% (w/v) BSA in 10 mM Tris–CL, 140 mM NaCl, 1.5 mM NaN_3_, pH 7.4) overnight at 4°C. The plate was then washed with washing buffer (TBS with 0.05% Tween 20 and 5 mM CaCl_2_). EDTA-plasma samples were thawed on ice and suspended in barbital buffered saline (BBS; 4 mM barbital, 145 mM NaCl, 2 mM CaCl_2_, 1 mM MgCl_2_, pH 7.4), to a final plasma concentration of 6%. Wells receiving only BBS buffer were used as negative controls. Plasma solutions were incubated on the coated plate at 37°C for 1 h 30 min (40 μL/well). The plate was washed and incubated for 1 h 30 min at RT with a polyclonal anti-human C3c antibody (Dako, A0062) diluted 1:5,000 in washing buffer. After washing, the plate was incubated with an alkaline-phosphatase labeled goat anti-rabbit IgG antibody (Sigma A-3812) diluted 1:5,000 in washing buffer for 1 h 30 min at RT. Following washing, the assay was developed by adding 100 μL substrate solution (Sigma Fast p-Nitrophenyl Phosphate tablets, Sigma). The absorption at OD405 nm was then measured using the Infinite M200 spectrofluorimeter managed by Magellan software (Tecan, CH).

### LP Initiator Quantification

Ficolin-1, ficolin-2, ficolin-3, and MBL in plasma were analyzed after plasma incubation on acBSA or mannan-coated plates prepared according to the procedure reported in the previous paragraph. The plates were incubated for 1 h 30min at RT with mouse polyclonal anti-human MBL (HM2061, Hycult Biotechnologies, The Netherlands), anti-human ficolin-1 (41), anti-human ficolin-2 (37), and anti-human ficolin-3 (HM2089, Hycult Biotechnologies, The Netherlands), all diluted 1:100 in washing buffer. After washing, the plates were incubated with an HRP labeled goat anti-mouse IgG antibody (Santacruz, CA, USA) diluted 1:1,000 in washing buffer for 1 h 30 min at RT. Following washing, the assay was developed by adding 100 μL substrate solution TMB (TMB Substrate Kit; code 34021; Thermo Scientific, MA, USA; 1:1 with H_2_O_2_ solution). The reaction was stopped by adding 100 μL H_2_SO_4_ 2M, and absorption at OD450 nm was measured as above reported. The specificity of ficolin binding to acBSA was shown by the lack of binding to BSA or to uncoated plates (Figure S2 in Supplementary Material).

### Immunofluorescence Quantification

Ficolin-2 stained sections were acquired at 10× (pixel size of 0.646 μm) with an Olympus BX-61 Virtual Stage microscope so to have complete stitching of the whole plaque for the two color channels (DAPI and ficolin-2). Analysis was done using Fiji software. Briefly, the region of interest was delineated within the necrotic area referring to the corresponding H&E image. After separation of the color channels, the ficolin-2 signal channel was normalized by imposing a 0 gray level value to background noise. To this purpose, three background areas were calculated for their mean gray value and this mean value subtracted to the whole image. Ficolin-2 signal was then calculated as integrated density within the region of interest.

### Blinding and Statistical Analysis

All histological and plasma quantifications were performed by investigators blinded to patients’ clinical information. Patients were stratified by the degree of carotid stenosis (≤75 vs. >75%) to assess whether the relationships between plaque components were modified by severity of atherosclerosis. Fisher’s exact test was used to define the association between hemorrhagic content and plaque rupture and whether patients with low stenosis (≤75%) had higher hemorrhagic plaques. Column analysis after patient stratification was done by Mann–Whitney test in case of non-normal distribution of data or unpaired *t*-test in case of normal distribution. Normal distribution was assessed by Kolmogorov–Smirnov test. A “vulnerability score” was obtained stratifying plaque components (hemorrhagic content, lipid core area, media thickness, and infiltrated content) into four groups according to their quartiles. The total score ranged from 4 (stable plaque) to 16 (vulnerable plaque). Correlation analysis was performed by computing a Pearson *r* or Spearman *r* depending on data distribution. The Forest plot was obtained using the odds ratios calculated stratifying patients for high plaque vulnerability (defined as a score ≥12) and high protein levels (defined as optical density ≥ median of each protein tested in the ELISA assay). Correlations of ficolin-2 integrated density were done by computing the Spearman *r* since ficolin-2 values did not have a normal distribution (Kolmogorov–Smirnov test for normality). Statistical analysis was performed using standard software packages GraphPad Prism (GraphPad Software Inc., USA, version 6.0). All tests were two-sided and *p* values lower than 0.05 were considered statistically significant. Details on the statistical analysis applied for each experiment are reported in figure legends.

## Results

### Patients’ Details and Stratification Strategy

Of the 42 patients undergoing endoarterectomy, 5 were excluded due to excessive plaque fragility hampering cryostat sectioning (4) or to unsatisfactory staining (1). We therefore analyzed a total of 37 patients (Table 1). The mean age of the patients was 73.03 ± 7.42. In line with the prevalence of atherosclerosis in the general population (42, 43), men outnumbered women (31 vs. 6). All patients but one received either statins or antiaggregants; therefore, we did not correct data for treatment. The cohort was homogeneous for conventional risk factors, with majority of either current or former smokers (73%), having hypertension (86%), and dyslipidemia (92%).

Only 30% (11 out of 37) of patients were symptomatic, e.g., had single or recurrent transient ischemic attacks, making them eligible for urgent surgery. Clinically, patients were classified according to stenosis degree (%) in five ranks: 70, 75, 80, 85, and 90%.

**Figure 2:**
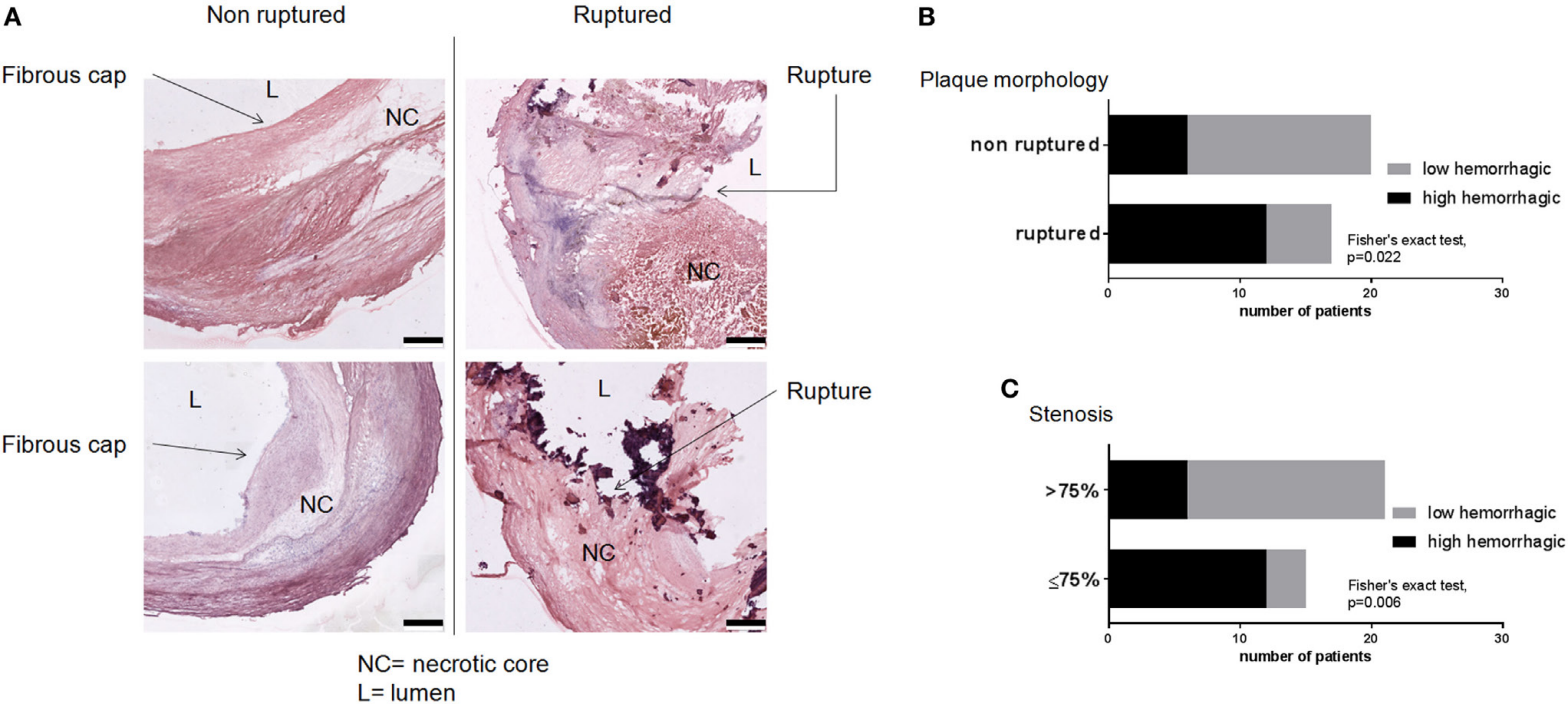
**Prevalence of ruptured and hemorrhagic plaques**. **(A)** Plaques were evaluated for the presence of rupture sites by an investigator blinded to the clinical and histological data. Scale bar = 500 μm. **(B)** Ruptured plaques were associated with high hemorrhagic content. **(C)** Plaques from LS patients (stenosis ≤ 75%) had higher hemorrhagic content indicating their vulnerability. Threshold between high and low hemorrhagic content was set at cohort’s median value (1.98%). Fisher’s exact test, *p* = 0.022 for **(B)** and *p* = 0.006 for **(C)**.

Seventeen out of 37 patients showed ruptured plaques according to the morphological evaluation proposed by Virmani et al. (36) and depicted in Figure 2A. Plaque rupture was associated with a high hemorrhagic content (>1.98%, threshold defined on the hemorrhagic content median value; Fisher’s exact test, *p* = 0.022, Figure 2B). We therefore used the measure of hemorrhagic content as a quantitative indicator of plaque rupture. Contingency analysis revealed that patients with less stenosis (70–75%) were more likely to have hemorrhagic plaques than patients with higher stenosis (80–85–90%, Fisher’s exact test, *p* = 0.006, Figure 2C). This suggests that in the cohort analyzed, patients with ≤75% stenosis had more ulcerated, rupture-prone plaques (44). On the contrary, stenosis was not associated with the occurrence of symptoms (Fisher’s exact test, *p* = 1.000, data not shown). The observation that in the analyzed cohort, patients with low degree of stenosis had ulcerated, rupture-prone plaques was used as a criteria to stratify patients according to the presence of vulnerable plaques (low stenosis, LS) vs. those with stable plaques (high stenosis, HS). To test the hypothesis that lower degree of stenosis may be due to the erosion of vulnerable plaques and to define a histology-based evaluation of plaque vulnerability, we next quantified parameters of plaque morphology.

### Histological Quantification of Morphological Characteristics Associated with Plaque Vulnerability

**Figure 3:**
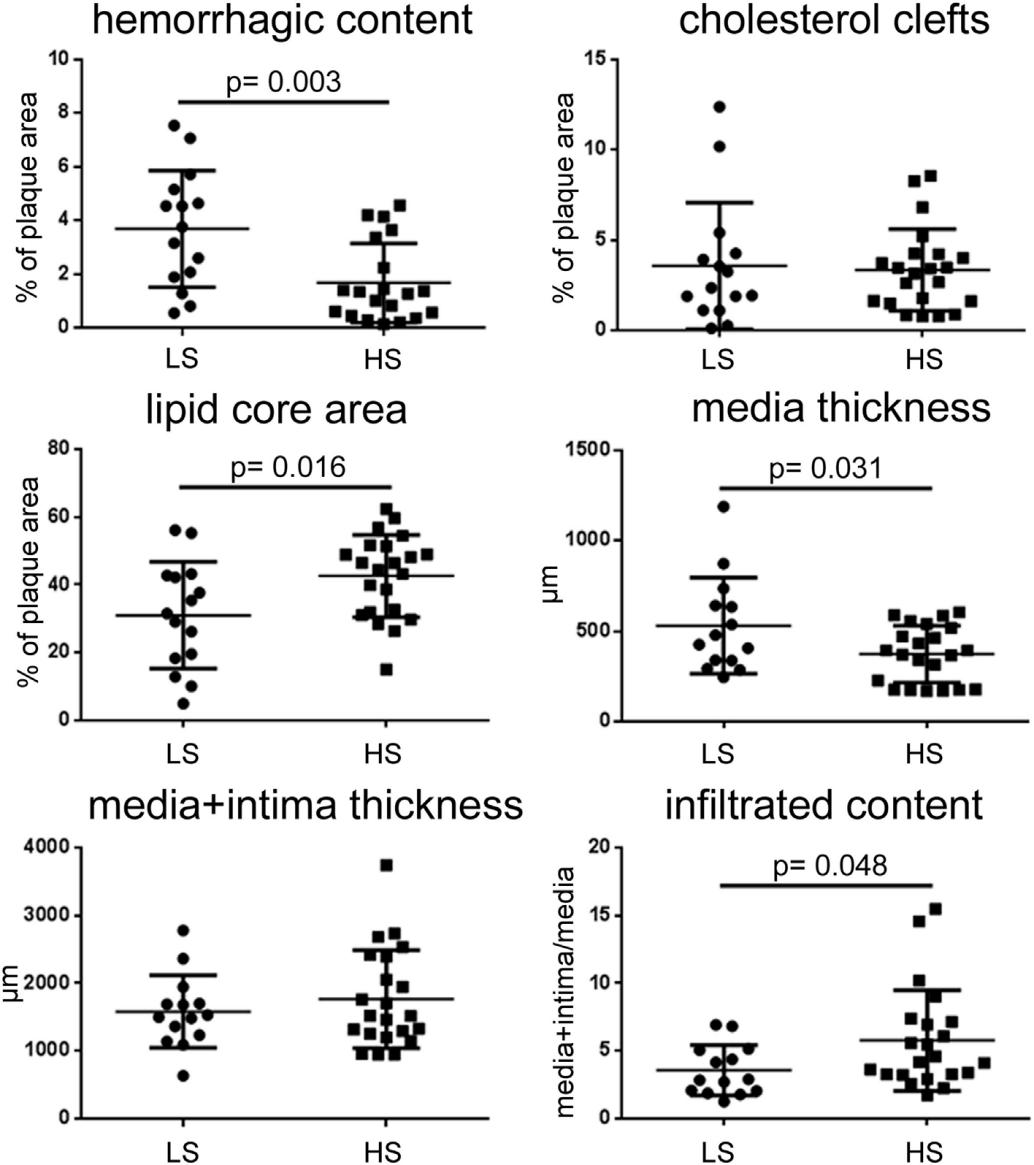
**Quantitative histological analysis of plaque morphology after patient stratification for stenosis**. LS patients had plaques with higher hemorrhagic content and media thickness, smaller lipid core area, and lower infiltrated content than HS. Thus LS plaques show erosion. Data presented as dot plots, line at mean ± SD. Mann–Whitney test for hemorrhagic content, unpaired *t*-test for others. LS, low stenosis; HS, high stenosis.

Atherosclerotic plaques were quantified for the following morphological characteristics, representing parameters associated with plaque instability (45): hemorrhagic content, cholesterol clefts, lipid core area, media thickness, total tunica thickness, and infiltrated content (Figure 3).

Plaques from LS patients had higher hemorrhagic content than those from HS patients (mean ± SD: 3.69 ± 2.17 vs. 1.67 ± 1.48% of plaque area, *p* = 0.003). These two groups did not differ for either cholesterol cleft area or tunica thickness (media + intima thickness). Patients with LS had smaller lipid core area (31.04 ± 15.72 vs. 42.60 ± 12.12% of plaque area, *p* = 0.016) and less infiltrated content (3.57 ± 1.87 vs. 5.77 ± 3.73 total tunica/media, *p* = 0.048) than HS patients. In line with these data, LS patients had thicker tunica media (530.1 ± 266.0 vs. 372.5 ± 156.6 μm, *p* = 0.031) since either less infiltration exerts less compression on this area or lamina becomes thicker and flaky, therefore favoring instability.

These data indicate that, in the cohort of patients analyzed, eroded plaques (associated to LS and increased hemorrhagic content) can be histologically defined based on smaller infiltration and increased tunica media thickness.

### LP Initiator Presence in the Atherosclerotic Plaques

**Figure 4:**
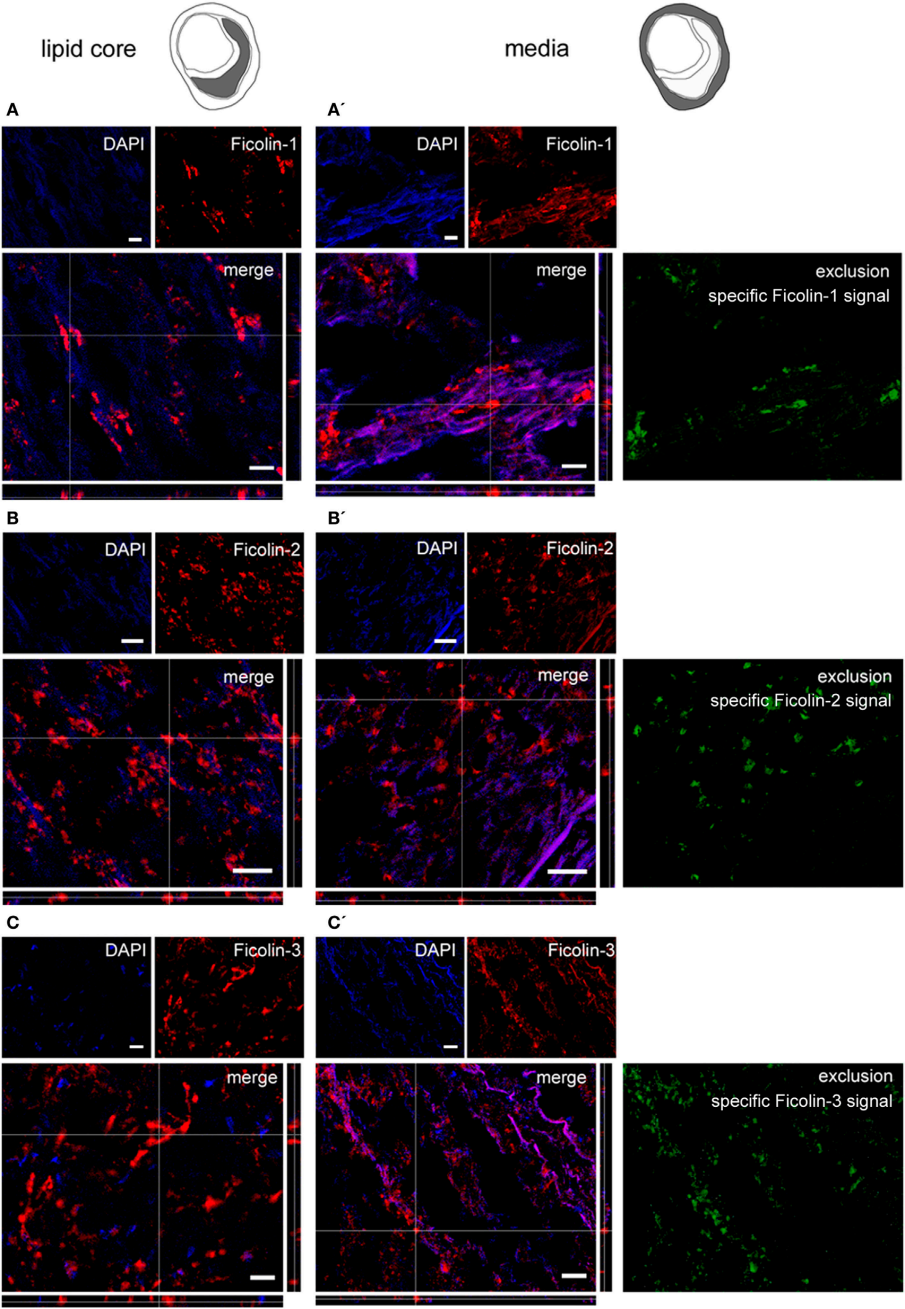
**Ficolin 1, 2, and 3 presence in plaques**. Microphotograms of the lipid core (left) and tunica media (right). **(A,A′)** Ficolin-1 (red) was in both lipid core and tunica media. To exclude the collagen non-selective fluorescent signal, we obtained an exclusion image showing only the ficolin-1 specific signal (green) and confirmed ficolin-1 presence in tunica media. **(B,B′)** Ficolin-2 (red) and **(C,C′)** Ficolin-3 (red) were also in lipid core and tunica media. Nuclei are blue. Scale bars = 20 μm.

**Figure 5:**
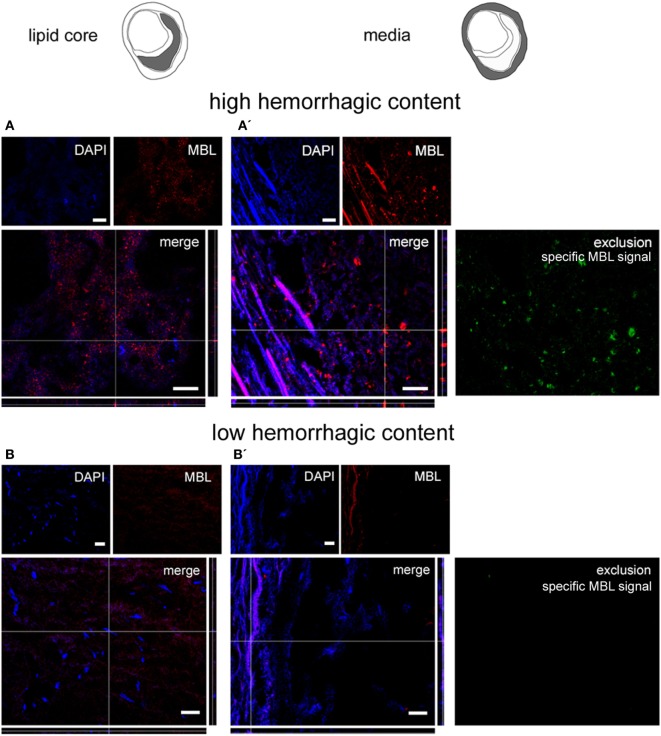
**Mannose-binding lectin (MBL) presence in plaques**. Microphotograms of the lipid core (left) and tunica media (right). MBL was present in lipid core and tunica media in high hemorrhagic [hemorrhagic content >1.98% **(A,A′)**] but not in low hemorrhagic **(B,B′)** plaques as further confirmed by the exclusion images. Nuclei are blue. Scale bars = 20 μm.

We next sought to detect the presence of LP initiators within the plaques. We performed immunofluorescence for ficolin-1, ficolin-2, ficolin-3, and MBL, initiators of the LP (46). Ficolin-1 (Figures 4A,A′), ficolin-2 (Figures 4B,B′), and ficolin-3 (Figures 4C,C′) were present both in lipid core and tunica media, as further confirmed by the exclusion images showing ficolin selective signal in tunica. Ficolins were found in plaques with either low or high hemorrhagic content. MBL was present in lipid core and tunica media in plaques with high hemorrhagic content (>median 1.98%, Figures 5A,A′), while did not show any presence in plaques with low hemorrhagic content (<1.98%, Figures 5B,B′).

### Plaque Vulnerability Score

**Figure 6:**
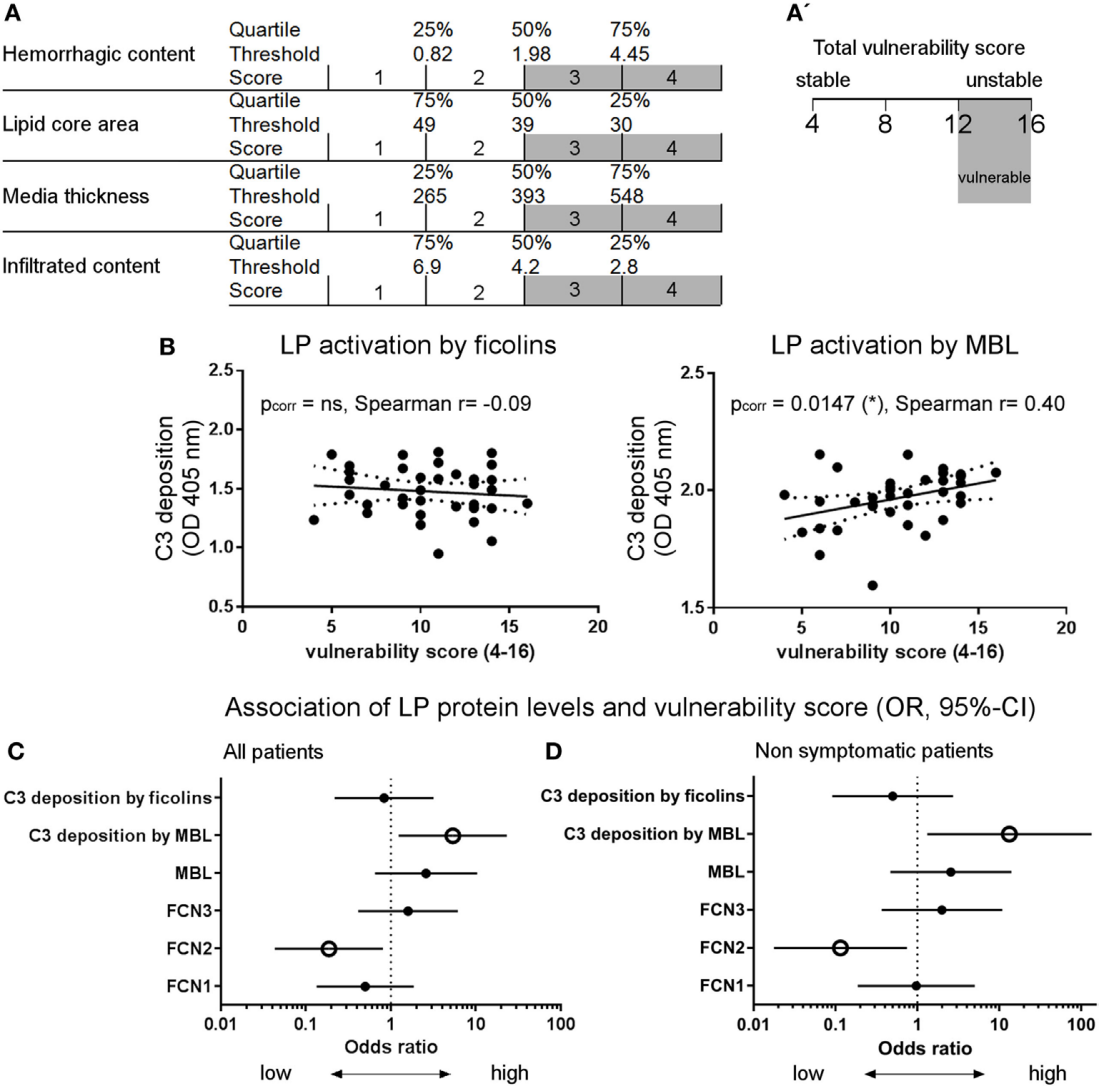
**Activation of the lectin complement pathway in atherosclerotic patients**. **(A)** Scheme for the definition of the vulnerability score obtained on: hemorrhagic content, lipid core area, media thickness, and infiltrated content. The score (1–4) for each parameter depends on the value distribution in a given quartile, thus the total score ranges from 4 (stable) to 16 (vulnerable). **(A′)** A total vulnerability score ≥12 was used as a cut-off for high vulnerability when computing the odds ratio (OR). **(B)** LP activation by mannose-binding lectin (MBL), but not by ficolins, positively correlated with the vulnerability score. Spearman’s correlation test. **(C)** Association (OR; 95% confidence interval) between LP and vulnerability score was significant when considering LP activation by MBL and ficolin-2 low plasma levels. OR was calculated based on high vulnerability [score ≥12, see **(A′)**] and high protein plasma levels (≥median OD). **(D)** Association between LP and vulnerability score in non-symptomatic patients only (26/37). In line with the whole cohort data, the association was significant when considering LP activation by MBL and ficolin-2 low plasma levels. OR was calculated based on high vulnerability (score ≥12) and high protein plasma levels (≥ median OD). Empty dots indicate *p* < 0.05.

The presence of ficolin-1, -2, and -3 and MBL in the atherosclerotic plaques suggested that the LP is activated and involved in atherosclerotic events, therefore making it a candidate marker of pathology. To correlate LP activity to plaque vulnerability, a comprehensive vulnerability score was obtained based on quartile distribution of all four risk parameters (Figure 3): hemorrhagic content, lipid core area, media thickness, and infiltrated content. The total score ranged between 4 (stable) and 16 (vulnerable, Figures 6A,A′). Table 2 summarizes the histological information relative to each degree of stenosis.

**Table 2:** **Histopathological data related to stenosis degree**.

	LS		HS		
	(70%)	(75%)	(80%)	(85%)	(90%)
Patients number (*n* = 37)	3	12	11	4	7
Ruptured (*n* = 17)	1	7	5	2	2
Not ruptured (*n* = 20)	2	5	6	2	5
Ruptured frequency	33	58	45	50	29
Vulnerability Score					
Hemorrhagic content	3.00	4.00	2.00	3.00	2.00
Lipid core area	3.00	3.00	2.00	1.00	3.00
Media thickness	3.00	3.00	2.00	1.00	3.00
Infiltrated content	4.00	2.50	2.00	1.00	3.00
Total Vulnerability Score	12.00	12.50	8.00	6.00	11.00

*LS, low stenosis; HS, high stenosis*.

### Measurement of Residual LP Activity and Proteins in Plasma Samples

**Figure 7:**
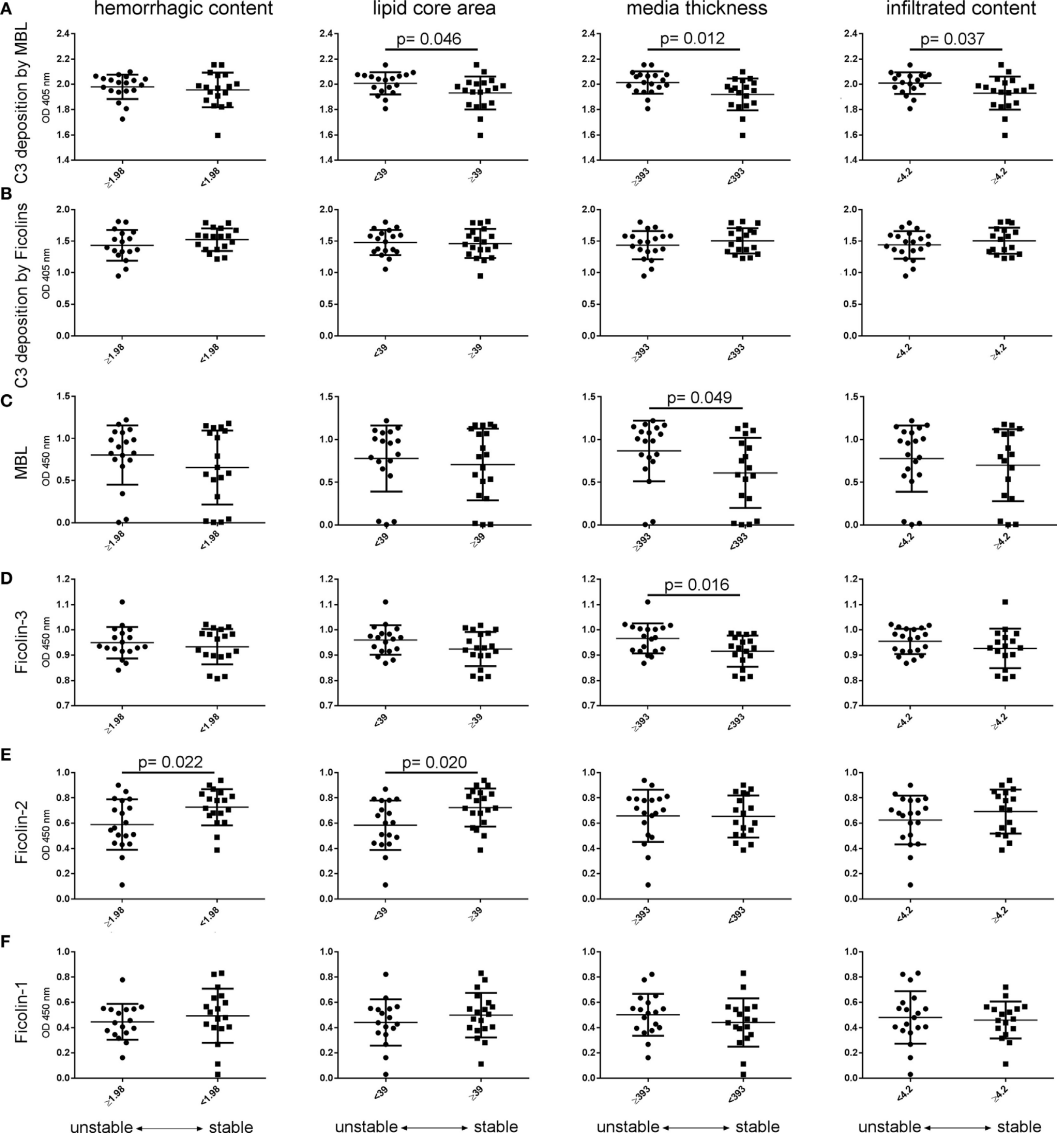
**Activation and initiators of the lectin pathway comparing patients with stable vs. unstable plaques**. Patients were stratified using the median values of the four histological parameters associated with plaque vulnerability, namely hemorrhagic content, lipid core area, media thickness, and infiltrated content (see Figure 3). **(A)** LP activation driven by mannose-binding lectin (MBL) (C3 deposition on mannan) increased in plasma from patients with low lipid core area, high media thickness, and low infiltrated content (unstable plaques). **(B)** In contrast, LP activation driven by ficolins (C3 deposition on acBSA) did not change between stratified patients. **(C)** MBL was higher in plasma from patients with increased media thickness as well as **(D)** Ficolin-3. **(E)** Ficolin-2 decreased in plasma of patients with high hemorrhagic content and low lipid core area (vulnerable plaques). **(F)** Ficolin-1 did not change throughout morphological stratifications. These observations are in line with the data presented in Figure 6C. Data are presented as dot plots with line at mean ± SD. In abscissae, median values for each parameter are indicated. All measures met normal distribution; therefore, unpaired *t*-test was performed, significant *p* values are indicated in graphs.

A functional ELISA was used to measure residual LP activity in plasma based on C3 deposition on plates coated with the best-affinity ligands for MBL (mannan) or ficolins (acBSA) (38), as indicated by the data shown in Figure S3 in Supplementary Material. Vulnerability score significantly correlated with LP activation by MBL (C3 deposition on mannan, Spearman’s correlation test, *p* = 0.015), but not by ficolins (Figure 6B). In line with this, the LP activation by MBL was associated with vulnerable plaques (Figure 6C) showing lower lipid core, increased tunica media thickness, and lower infiltrated content (Figure 7A). In contrast, ficolin-2 was decreased in plasma from patients with vulnerable plaques, indicating its use (consumption) in pathway activation and possibly driving plaque instability (Figure 6C). However, ficolin-2 plasma level changes did not affect the functional assay of C3 deposition on acBSA (Figures 6C and 7B).

It is likely that an acute cardiovascular event (such as TIA, these patient symptom) affects the circulating levels of the LP proteins (17, 19). This implies that a central event may affect LP levels independently from the atherosclerotic process. In order to have a clear view of the events related to atherosclerosis, we have analyzed our data excluding the symptomatic patients (Figure 6D; Figure S4 in Supplementary Material). The associations between LP protein plasma levels and plaque vulnerability were confirmed analyzing only the non-symptomatic patients.

By analyzing the plasma levels of each LP initiator, stratifying patients using the median values of the four histological parameters associated with plaque vulnerability—hemorrhagic content, lipid core area, media thickness, and infiltrated content—we found that MBL and ficolin-3 were higher in plasma from patients with increased media thickness (Figures 7C,D). Ficolin-2 decreased in plasma of patients with high hemorrhagic content and low lipid core area (vulnerable plaques, Figure 7E). Ficolin-1 did not change throughout morphological stratifications (Figure 7F). These observations are in line with the Forest plot data presented in Figure 6C.

**Figure 8:**
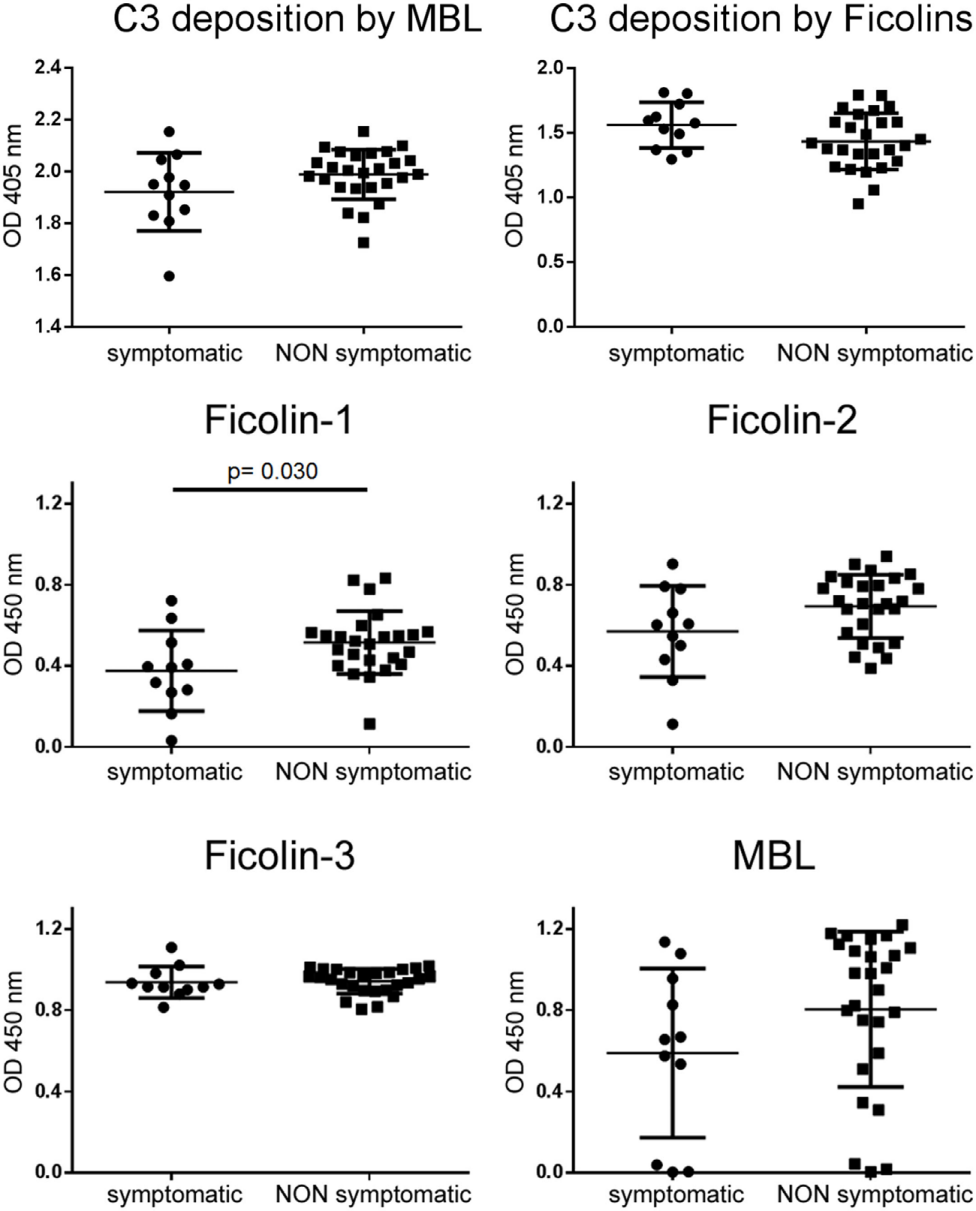
**LP activation and initiator plasma levels after patient stratification on symptomatology**. C3 deposition driven by either MBL or ficolins did not change depending on symptomatology. Ficolin-1 was significantly lower in plasma of symptomatic patients. Data presented as dot plots with line at mean ± SD. Unpaired *t*-test, significant *p* values are reported.

When we stratified patients for symptomatology, plasma levels of ficolin-1 were lower in symptomatic than in asymptomatic patients (Figure 8), and no other differences were found in the other LP initiators.

### Measurement of Ficolin-2 Intraplaque Deposition

**Figure 9:**
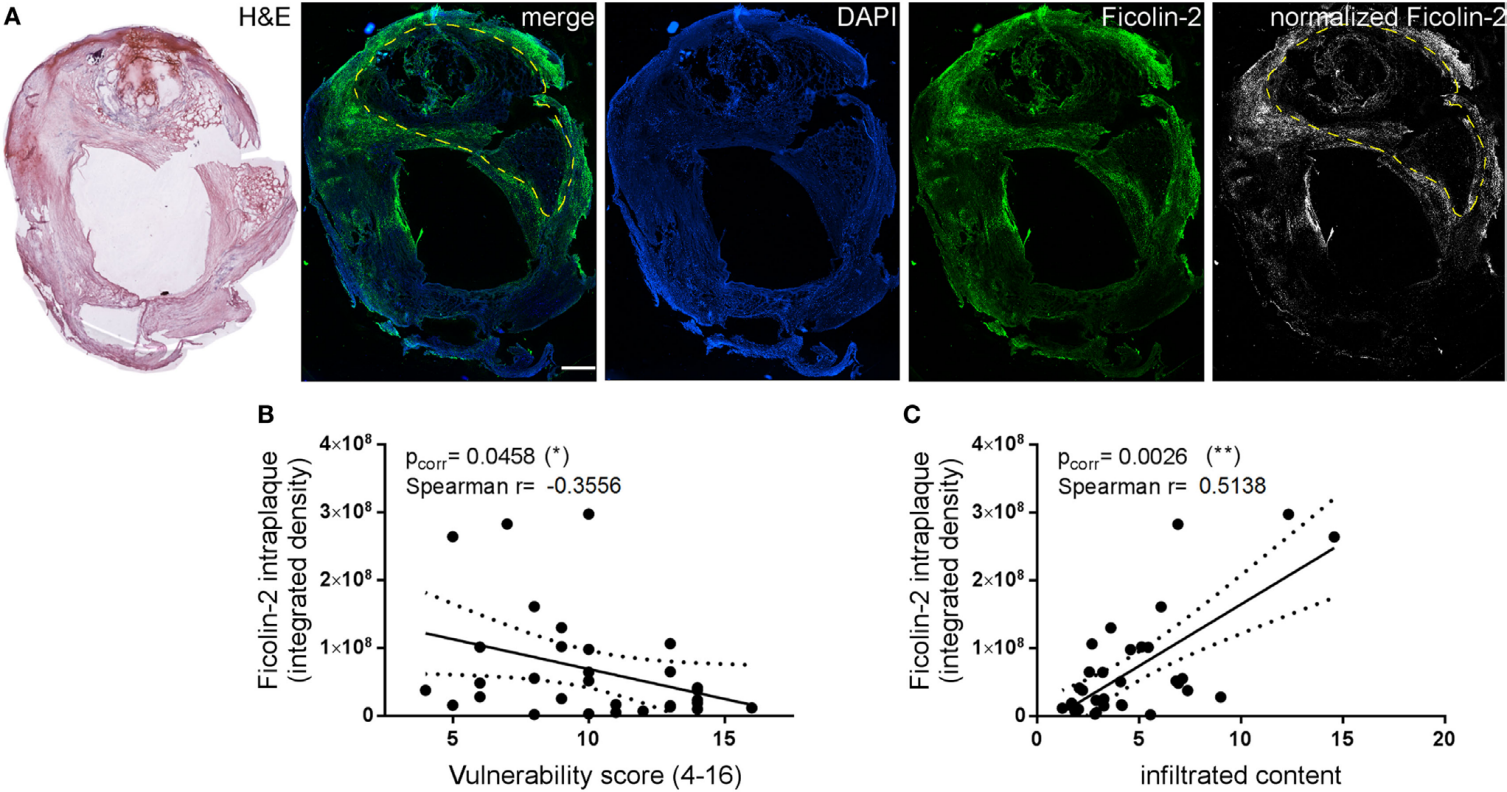
**Analysis of ficolin-2 intraplaque presence**. **(A)** Ficolin-2 intraplaque presence was calculated by acquiring the whole plaque and obtaining the ficolin-2 specific signal after normalization with background subtraction. The region of interest to be analyzed (necrotic area, yellow dashed outline) was delineated referring to the corresponding hematoxyline and eosin (H&E) image. Scale bar = 1 mm. **(B)** Ficolin-2 intraplaque levels were inversely correlated to the vulnerability score. **(C)** On the contrary, ficolin-2 intraplaque levels were directly correlated with the infiltrated content. Spearman correlation test, *p* values indicated in the figure.

To define the correlation between LP protein deposition in the plaque and plaque vulnerability, we stained the plaques from all patients for ficolin-2, the only LP initiator protein changing in association with the vulnerability score (see Figures 6C,D). We acquired the whole plaque at high resolution and normalized the color channel with the ficolin-2 signal (Figure 9A). Ficolin-2 intraplaque levels (expressed as integrated density of fluorescent pixels in the region of interest), calculated in the necrotic core, were inversely correlated with the vulnerability score (Figure 9B), similarly to what is observed with circulating ficolin 2. Ficolin-2 intraplaque levels were directly correlated with the infiltrated content (Figure 9C).

## Discussion

This study demonstrates the potential use of LP components as markers for cardiovascular risk in atherosclerotic patients showing that: (1) ficolins and MBL, initiators of the LP activation, are present within the lipid core and the tunica media of atherosclerotic plaques; (2) plasma levels of ficolin-2 are decreased in patients with vulnerable plaques and those of ficolin-1 are decreased in symptomatic (vs. non-symptomatic) patients experiencing a transient ischemic attack; (3) the LP activity driven by MBL is increased in plasma of patients with vulnerable plaques.

We first defined histological parameters allowing to identify vulnerable, rupture-prone plaques within the cohort of analyzed patients. We stratified patients in two groups based on the degree of stenosis, namely LS and HS, as assessed by preoperative echocolordoppler. Plaques from LS patients had increased hemorrhagic content, measured by histological quantification, than those from HS patients, indicating their unstable nature. In this regard, we here report the association of hemorrhagic content with signs of rupture within the plaque. Moreover, intraplaque neovascularization has been associated with increasing plaque inflammation and vulnerability (47, 48), and microvessel-related intraplaque hemorrhage has been reported as a predictor factor for cardiovascular events (49, 50). Intraplaque hemorrhage therefore indicates plaques subjected to erosion, which are classifiable as unstable (45). Eroded plaques are less likely to cause vessel narrowing compared to stable, fully developed plaques, explaining their prevalence in mildly stenotic patients (51). Along with increased intraplaque hemorrhagic content, plaques from LS patients have smaller lipid core and lower intimal infiltration. Moreover in these rupture-prone plaques the tunica media is thicker than that from HS patients, indicating a compensatory enlargement of the vessel segment. The complex transition from compensatory enlargement to plaque disruption may be a fine interaction between progressive intimal disease and evolving pathology at the intimomedial interface, including rupture of the internal elastic lamina and medial inflammation (51).

We then defined four morphological parameters, namely hemorrhagic content, lipid core area, infiltrated content, and tunica media thickness that are associated with plaque instability in the analyzed cohort of patients. Our findings are partially in line with the observation by Takaya et al. who correlated the presence of intraplaque hemorrhage and the larger maximum wall thickness with the risk of cerebrovascular events in 154 asymptomatic patients with definitive 50–70% stenosis, followed-up for 12 months (52).

We next explored whether histologically defined vulnerable plaques were associated with LP activation, aiming at identifying early markers of cardiovascular risk. Evidence supports the involvement of the complement system in atherosclerosis. When the vascular endothelium is injured, the entrance of lipids and active complement fragments, as well as inflammatory cell recruitment into the media layer, initiates and intensifies atherosclerosis. Of the pathways of complement system activation, the classical and alternative have a dual function in atherosclerosis, counteracting plaque formation through debris clearance or favoring atherogenesis (12). Less is known on the role of the LP that may have either an anti-atherogenic (24–27) or a pro-atherogenic (29–31) and pro-thrombotic function (12).

We analyzed the presence of ficolins and MBL in the atherosclerotic plaques. We report the presence of ficolin-1, ficolin-2, and ficolin-3 in carotid plaques, specifically within the lipid core and in the collagen-rich tunica media. Füst and collaborators (17) showed increased ficolin-2 and ficolin-3 serum levels in asymptomatic patients with severe carotid atherosclerosis compared to healthy controls or to patients after acute stroke. This suggests the involvement of ficolin-2 and ficolin-3 in the atherosclerotic process and their rapid consumption after an acute event. We show a significant association between low ficolin-2 plasma levels and plaque vulnerability, indicative of ficolin-2 use, as the plaque becomes vulnerable, a risk factor for an acute cardiovascular event. Interestingly, the levels of intraplaque deposited ficolin-2 are inversely correlated with plaque vulnerability, in line with what is observed with circulating ficolin-2. Ficolin-2 deposited in the plaque correlates directly with the infiltrated content. Based on these observations, as a working hypothesis, we can propose that ficolin-2 contributes to plaque erosion, an event that evolves over time before the patients’ surgery and that we can observe only at a late stage (post-surgery) when intraplaque ficolin-2 is lost as a consequence of erosion and circulating ficolin-2 is low since it is consumed, i.e., used for the pathway activation. As an alternative hypothesis ficolin-2, whose high intraplaque levels correlate with low plaque vulnerability, may be protective. In this case, ficolin-2 could contribute to debris clearance, in line with its function as an opsonin for phagocytosis independent of complement activation (53).

Our data originally show ficolin-1 presence within atherosclerotic plaques and its significantly lower levels in symptomatic patients with transient ischemic attacks compared to non-symptomatic. This observation is in line with a previous work by our group reporting that ficolin-1 is a sensitive prognostic marker for stroke patients (19), and further implicates ficolin-1 in acute brain injury.

Reports on MBL in atherosclerosis are contrasting. Patients with MBL deficiency have higher risk to develop a cardiovascular event after infection by *Chlamydia pneumoniae* (25). Similarly, patients affected by systemic lupus erythematosus and homozygous for MBL variants develop an increased risk of arterial thrombosis (26). In a cohort of American Indians, variant MBL genotypes have been associated with increased risk of coronary artery disease (27). In line with these data, Vengen et al. showed that the presence of MBL variant allele may increase the risk of myocardial infarction in young individuals (32) and Saevarsdottir proposed for MBL an anti-atherogenic role (28). On the other hand, high MBL serum levels are associated with increased risk of coronary artery disease in men (30) and in diabetic patients (29). Moreover, patients with normal MBL genotype are more likely to experience restenosis after endarterectomy (31).

We here show that MBL is selectively present in ulcerated (highly hemorrhagic) plaques, maybe due to its limited ability to extravasate (15). By the functional *in vitro* assay measuring C3 deposition on mannan, our study suggests that MBL-driven LP activation is associated with plaque instability. This association is further supported by the observation that MBL is present only in ulcerated plaques undergoing rupture, therefore extending previous work reporting MBL binding to intraplaque cholesterol crystals (53), clearly defining that plaque rupture is needed for MBL entry. Thus, although alternative interpretations cannot be excluded, i.e., that MBL is not consumed due to a lack of involvement in the condition, the overall data may indicate the association of MBL with plaque instability. This observation extends the concept that MBL in atherosclerosis may have different effects depending on time in the disease process and the specific clinical setting (29, 32).

In the analyzed cohort, 30% of patients were symptomatic, i.e., they experienced a TIA and were eligible for urgent surgery. When we compared symptomatic vs. non-symptomatic patients, we could not observe any significant difference in the morphological parameter, possibly because the groups were not balanced. However, we could detect significantly lower levels in circulating ficolin-1 implying that the symptomatology can influence LP protein levels. In order to test whether LP protein plasma levels may be markers of plaque vulnerability independently from symptomatology—their use should ideally help decision-making of surgeons in asymptomatic patients—we repeated the analysis excluding the 11 symptomatic patients. The data obtained are consistent with those obtained on the whole cohort indicating that LP protein plasma levels are reliable peripheral markers of plaque vulnerability, independently from symptomatology.

We cannot exclude that LS patients have still growing rather than eroding plaques. However, in the analyzed cohort of patients, LS is strongly associated with the presence of high intraplaque hemorrhagic content, a clear index of erosion as confirmed by its association with plaque rupture. Of note, LS is not associated with symptomatology meaning that the observed changes in LP activation (C3 deposition on mannan), and in ficolin-2 plasma levels in patients with vulnerable plaques are not a mere consequence of a cardiovascular event, but rather anticipate clinical symptomatology.

Potential limitations of this study include the small size of patients’ cohort that hampered stratifications based on age, sex, or relevant comorbidities, all potentially impacting on plaque morphology (43, 54). However, the cohort that we analyzed reflects the epidemiology of patients suffering from significant atherosclerosis and eligible for surgical intervention (54). Data on the LP activation need to be extended by recruiting more patients and following them before and after surgery. Moreover the use of combinatorial approaches including advanced imaging techniques and assessment of other plasma complement proteins could add details on the LP role in atherosclerosis. This will help address the validity of the LP as a marker for future cardiovascular risk in atherosclerotic patients, offering solid reasons to select timely treatments in clinical practice.

## Author Contributions

SF, CP, and RZ designed the work, performed the analysis, interpreted the data, and drafted the manuscript. AF and FN designed the work, collected the patients’ clinical data and samples, interpreted the data, and drafted the manuscript. DB and MO performed the analysis, interpreted the data, and revised the manuscript. FS, PG, and M-GS designed the work, interpreted the data, and drafted the manuscript. All authors have given final approval of the current version and agree to be accountable for all aspects of the work in ensuring that questions related to the accuracy or integrity of any part of the work are appropriately investigated and resolved.

## Conflict of Interest Statement

The authors declare that the research was conducted in the absence of any commercial or financial relationships that could be construed as a potential conflict of interest.

## Abbreviations

HS, high stenosis (>75%); LP, lectin pathway of complement activation; LS, low stenosis (≤75%); MBL, mannose-binding lectin.
