# Supplementary material for: Lectin Pathway of Complement Activation Is Associated with Vulnerability of Atherosclerotic Plaques
Source: Front Immunol. 2017 Mar 16;8:288. doi: 10.3389/fimmu.2017.00288 (PMC5352714; doi:10.3389/fimmu.2017.00288)
Supplement: Supplementary file 1 [file Data_Sheet_1.PDF]

Supplementary figures

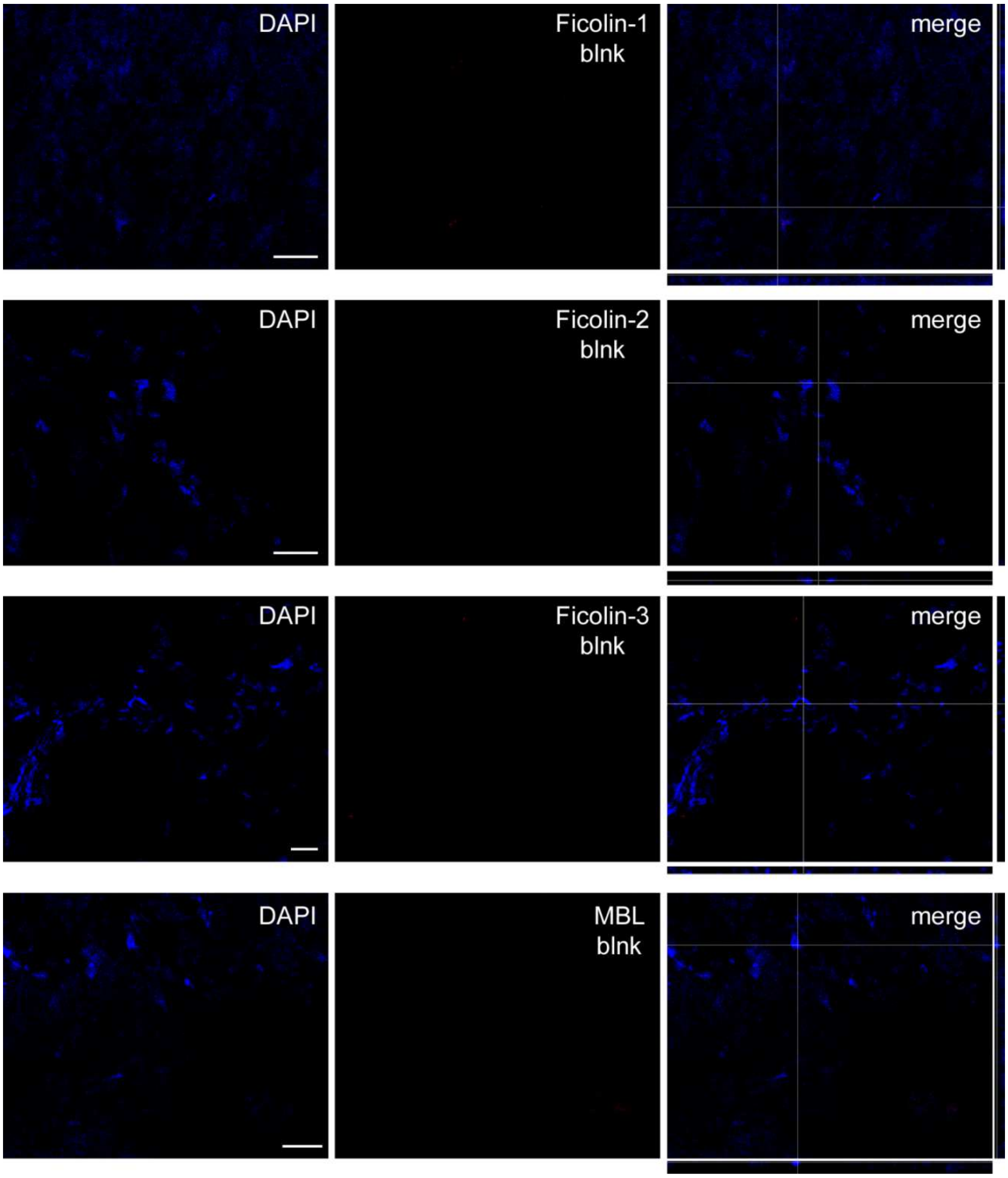

Figure S1. Negative controls for immunofluorescence stainings.
All staining procedures for ficolins and MBL did not yield any signal when the primary antibody was omitted. Bars = 20 µm.

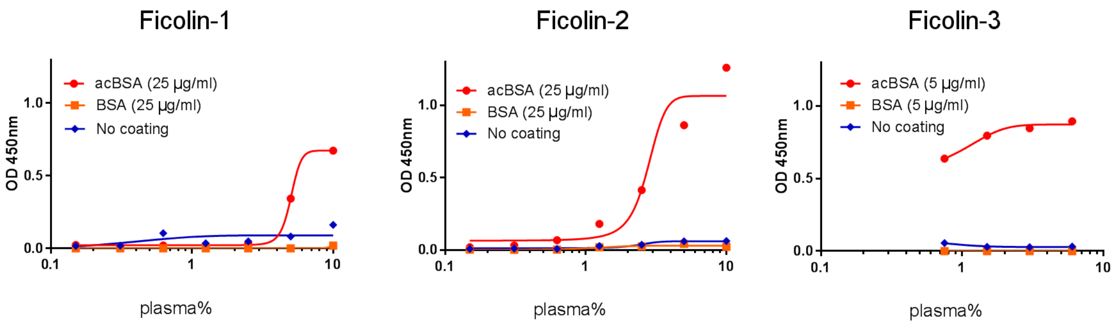

Figure S2. Experimental controls for the specificity of the ficolins' deposition assays.
Plates were coated with acBSA, BSA or non coated. Plasma at different dilutions was incubated following the protocol indicated in material and methods. For each ficolin a dilution-dependent signal was obtained only on acBSA coated plates. BSA or non coated plates gave no signal. Of the dilutions tested here, we chose 6% to perform the experiments reported throughout the study.

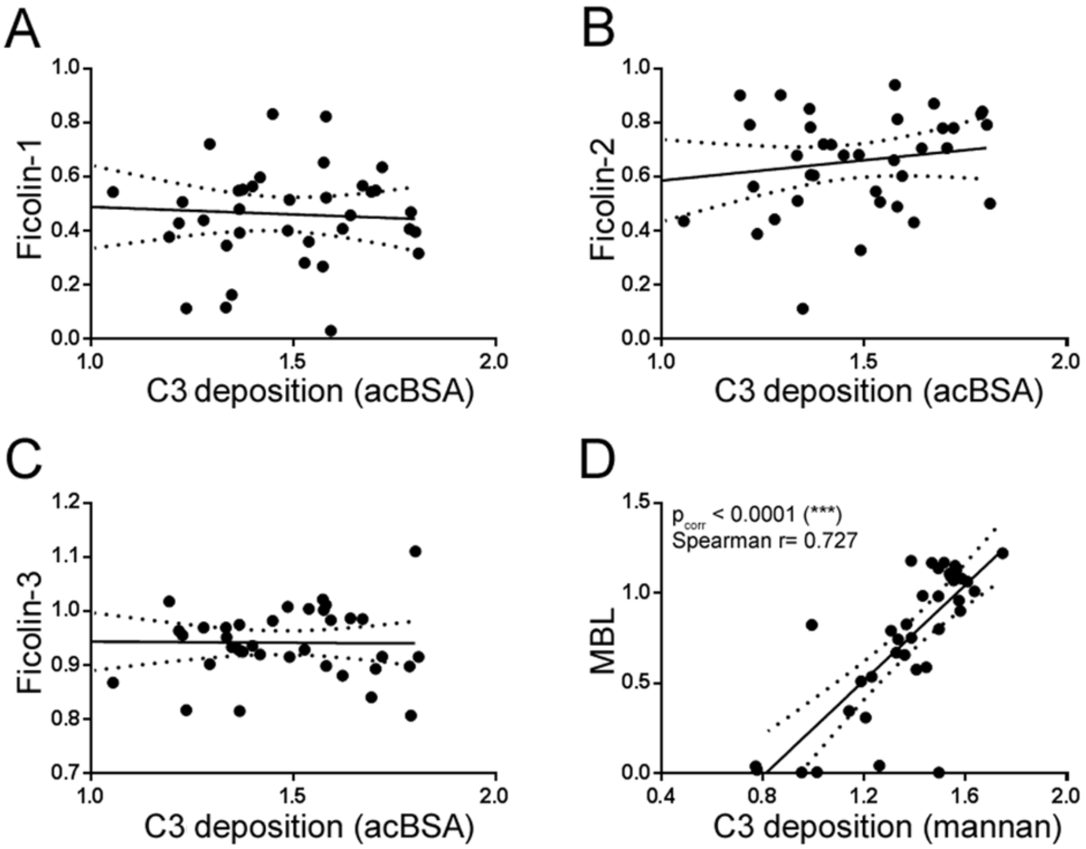

Figure S3. Correlations between C3 deposition (LP functional assay) on acBSA or mannan and ficolin or MBL levels.
The deposition of C3 on mannans depends exclusively on MBL, while that on acBSA on a global effect of all the three ficolins. To verify this in our experimental conditions, we correlated C3 deposition on different substrates and LP initiator levels. Ficolin-1 (A), -2 (B) and -3 (C) levels did not correlate with C3 deposition on acBSA (25 µg/mL). This deposition depends on all the three ficolins, whose levels were either increased or decreased in plasma samples (see figure 6C), thus supporting the overall lack of correlations with C3 deposition on acBSA. In contrast, C3 deposition on mannans which depends exclusively on MBL showed a strong correlation with plasma levels (D).

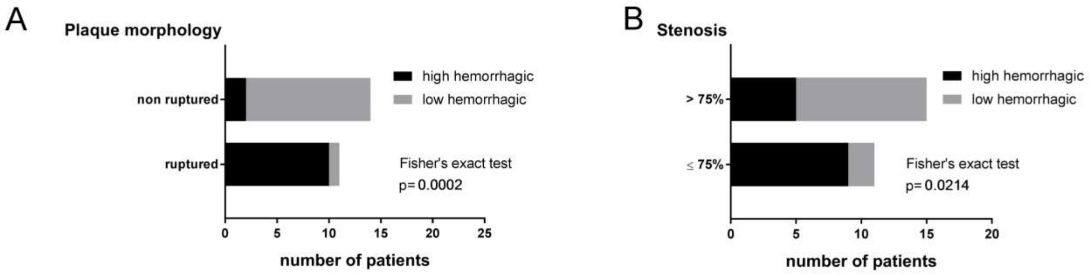

Figure S4. Analysis of prevalence of ruptured and hemorrhagic plaques in non symptomatic patients.

Patients were analyzed also excluding symptomatic patients (11/37). A) Ruptured plaques were associated with high hemorrhagic content. B) Plaques from LS patients (≤75% stenosis) had higher hemorrhagic content indicating their vulnerability. Threshold between high and low hemorrhagic content was set at cohort's median value (1.98%). Fisher's exact test, p= 0.0002 for A and p= 0.0214 for B.
